# Brolucizumab for Choroidal Neovascular Membrane with Pigment Epithelial Tear and Subretinal Fluid

**DOI:** 10.3390/jcm10112425

**Published:** 2021-05-30

**Authors:** Alper Bilgic, Laurent Kodjikian, Shail Vasavada, Shyamal Jha, Samaresh Srivastava, Aditya Sudhalkar, Thibaud Mathis

**Affiliations:** 1 Department of Retina, Alphavision Augenzentrum, 27568 Bremerhaven, Germany; drbilgicalper@yahoo.com; 2 Service d’Ophtalmologie, Centre Hospitalier Universitaire de la Croix-Rousse, Hospices Civils de Lyon, Université Claude Bernard Lyon 1, 69004 Lyon, France; laurent.kodjikian@chu-lyon.fr (L.K.); thibaud.mathis@chu-lyon.fr (T.M.); 3 UMR-CNRS 5510, Matéis, Villeurbane, 69100 Lyon, France; 4 Raghudeep Eye Hospital, Ahmedabad 380052, India; shail@raghudeepeyehospital.com (S.V.); shyamal@raghudeepeyeclinic.com (S.J.); samaresh@raghudeepeyeclinic.com (S.S.); 5 MS Sudhalkar Medical Research Foundation, Baroda 390001, India

**Keywords:** age-related macular degeneration, anti-vascular endothelial growth factor, brolucizumab, epithelial tear, exudation, optical coherence tomography

## Abstract

The aim of this study was to determine the utility of brolucizumab in the management of choroidal neovessels (CNV) with a retinal pigment epithelial (RPE) tear and subretinal fluid. We used a case series of patients with CNV who developed an RPE tear either spontaneously or following an intravitreal injection. All patients received intravitreal brolucizumab as primary or switch therapy. Appropriate data were collected. Follow-up was one year. The paired t-test was used to determine the significance of the results. The primary outcome measure was the change in best corrected visual acuity (BCVA). Secondary outcome measures were the change in subretinal fluid and complications, if any. A total of five patients were included in the analysis. The age range was 67−74 years and baseline BCVA was from 20/80 to 20/100. On average, all patients showed improvement in BCVA (*p* = 0.012) and also showed a significant anatomical improvement (*p* = 0.03). None of the patients had any complications, and all patients responded to additional anti-VEGF injections. In conclusion, all patients showed significant visual and anatomical improvement with brolucizumab; no complications were noted. All patients, including those who received switch, demonstrated a favorable anatomical and visual response to intravitreal brolucizumab without safety concerns.

## 1. Introduction

Retinal pigment epithelial (RPE) tears can occur in certain ocular conditions spontaneously or with intervention [1,2,3,4]. The visual prognosis depends largely, but not entirely, on the precise location of the scrolled-up RPE margin [1,3]. If subfoveal, it can lead to permanent visual dysfunction. Histopathological evidence shows that choroidal neovessels (CNV) may involve an area much wider than what is apparent clinically, and CNV activity can continue even if there is a tear [4]. The size of the pigmented epithelial detachment (PED) and a high number of intravitreal therapies are known risk factors of this complication [5]. In the event of an RPE tear, the subretinal fluid (SRF) enters the subretinal space, and intravitreal injections of anti-vascular endothelial growth factor (VEGF) agents are generally prescribed to aid its resolution [1]. Currently, intravitreal anti-VEGF therapy is considered the best recourse in such a situation [1]. However, limited literature works are available on the management of patients with CNV who develop RPE tears and have SRF.

The HAWK and HARRIER studies established the non-inferiority of the new molecule brolucizumab vis-a-vis aflibercept, with some analyses suggesting a superior anatomic outcome [6]. Brolucizumab is a single-chain antibody fragment (scFv), which is the smallest functional unit of an antibody. The small size of the molecule allows the delivery of a higher molar dose compared with larger molecules such as ranibizumab, aflibercept and bevacizumab. Brolucizumab was developed by grafting regions of an anti-VEGF antibody to a human scFv scaffold.

Nearly 50% of enrolled patients could receive 12 weekly injections, considerably reducing the treatment burden. However, concerns about safety with special reference to intraocular inflammation and vasculitis dampened the initial enthusiasm for the drug [7]. As data continue to evolve, the risk of serious adverse events is currently pegged at 4.6% [8,9].

This small case series looks at patients with CNV and RPE tears who received intravitreal brolucizumab as primary or switch therapy.

## 2. Materials and Methods

This was a retrospective, observational study conducted at the Alphavision Augenarzt Praxis, Bremerhaven, Germany. All patients with a diagnosis of RPE tear treated by brolucizumab were included. Treatment-naive eyes, as well as patients already treated with intravitreal injections, were included. We recorded the best corrected visual acuity (BCVA), and details of the anterior and posterior segment exam as well as the systemic examination and special investigations such as fluorescein angiography (and indocyanine green angiography where necessary), autofluorescence imaging (both using the Zeiss Visucam 450, Zeiss Meditec, Dublin, CA, USA) and optical coherence tomography raster scans using a spectral domain OCT (Cirrus, Zeiss Meditec, Dublin, CA, USA). This study complied with the tenets of the Declaration of Helsinki and was approved by an international review board (Ethics Committee of the French Society of Ophthalmology, IRB 00008855 Société Française d’Ophtalmologie IRB#1). Patients gave their informed consent to participate in the study.

All patients were given at least three intravitreal anti-VEGF injections each a month apart and monitored for BCVA, fluid status and RPE tears. In the case of switch therapy, patients were considered for change of anti-VEGF agents after at least 3 consecutive monthly anti-VEGF agents. Standard guidelines as elaborated upon in the HAWK and HARRIER trials were followed with respect to intravitreal brolucizumab therapy. Intravitreal injections were administered using a standardized aseptic technique.

Follow-ups were scheduled on post-intravitreal injection days 1 and 7 and then monthly for one year.

The primary outcome measure was the change in BCVA with treatment. The secondary outcome measures were the reduction in SRF and the complications, if any. Statistical analysis was performed using the paired t-test. Statistical significance was set at *p* < 0.05.

## 3. Results

We included five patients (five eyes) with CNV who developed an RPE tear either spontaneously (three patients) or during the course of intravitreal therapy (two patients). There were three males. All patients were free of systemic disease. The demographic characteristics and the baseline and final BCVA values are listed in Table 1.

Three patients had a mixed (type 1 + type 2) CNV; the other two had type 1 CNV as noted on fluorescein angiography. All patients showed significant visual and anatomical improvement until the end of the follow-up period. All patients had received at least five injections of brolucizumab until the last follow-up. All patients had a grade 3 tear.

The age range was 67−74 years and baseline BCVA was from 20/200 to 20/100. On average, all patients showed improvement in BCVA (*p* = 0.012) and also showed significant anatomical improvement (*p* = 0.03). None of the patients developed any complication nor did any patient require a switch of anti-VEGF therapy.

A total of three patients were treatment-naïve and two patients were previously treated with intravitreal injections of aflibercept. They were switched to aflibercept due to persistent fluid in the retina. These two patients showed BCVA improvement as well as retinal fluid regression.

**Table 1:** Important characteristics of patients with pigment epithelial tears who received brolucizumab.

Patient Age(y)/Sex	Baseline VA (Snellen)	Therapy Status (*n*: Number of Injections)	PED Height (µm) Prior to Tear	Central Macular Thickness (µm) Baseline	SRF after RPE Tear (µm)	Number of Brolucizumab Injections *	Central Macular Thickness (µm) 1 Year	VA at 1 Year
74/M (Figure 1)	20/160	Switch (4 AFB)	225	328	289	2	275	20/100
69/M	20/200	Treatment-naive	279	389	296	4	268	20/50
67/F	20/120	Treatment-naive	342	367	272	2	282	20/80
72/M	20/100	Treatment-naive	328	402	301	2	259	20/50
68/F	20/100	Switch (5 AFB)	303	331	277	3	272	20/70

AFB—aflibercept, F—female, M—male, PED—pigment epithelial detachment, SRF—subretinal fluid, RPE—retinal pigment epithelium, VA—visual acuity, y—year. * Indicates number of injections required to achieve complete resolution of fluid in all compartments. All patients received therapy as per HAWK and HARRIER protocols.

**Figure 1:**
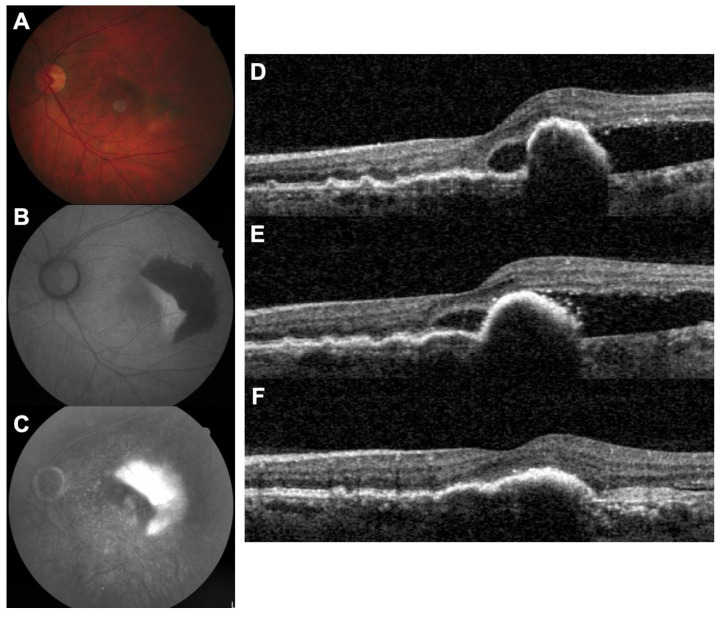
RPE tear complicating a type 1 CNV of a 74-year-old man (case 1). (**A**). Fundus photography showing RPE hyperpigmentation in the foveal area; (**B**). fundus autofluorescence demonstrating a large area of hypoautofluorescence in the temporal macula; (**C**). fluorescein angiography showing a window effect in front of the RPE tear; (**D**). baseline OCT showing a double layer sign under the fovea and a large PED with an absence of RPE in the temporal macula (i.e., RPE tear). SRF is present. (**E**). OCT after 4 monthly intravitreal injections of aflibercept, showing persistent SRF; (**F**). OCT after 2 intravitreal injections of brolucizumab showing a favorable response.

## 4. Discussion

This small case series shows good anatomical and functional results with intravitreal brolucizumab in patients with CNV and RPE tear with active leak. A total of three patients were treatment-naïve and two patients received switch therapy from aflibercept to brolucizumab. Both patients who received switch therapy showed further improvement in vision from baseline (at the time of switch). No side effects or complications, either as a consequence of the disease process or the administered therapy, were noted until the end of the follow-up period. None of the patients had a recurrence until the end of the follow-up period.

RPE tears [1,2,3,4] tend to eventually have a poor prognosis because of subretinal fibrosis, especially if the tear folds and encroaches upon the foveal center. In the event, it is necessary to ensure adequate therapy and achieve complete resolution of the associated fluid, thereby minimizing the detrimental effect of the condition on the retinal tissue.

The role of brolucizumab in the treatment of the exudative form of age-related macular degeneration (AMD) has been established through landmark Phase III trials. The smaller size of the brolucizumab molecule means a larger concentration of the drug can be delivered to the posterior segment of the eye via intraocular injection. This probably accounts for the improved efficacy and durability. On the other hand, as is true for any biological agent, it may account for a higher incidence of hypersensitivity such as a reaction.

There have been reports of adverse events with brolucizumab [7]. Notwithstanding, the drug is a useful alternative to currently available anti-VEGF agents for exudative AMD and, indeed, can eventually be the drug of choice given its potency, especially to reduce the treatment burden. More data will be available for perusal as the drug steadily penetrates global markets.

With just five cases to report, we have limited evidence on the efficacy and safety of brolucizumab in this particular subgroup of patients with exudative AMD, although none of the patients developed any complications. Further trials will be necessary to provide conclusive evidence. We suggest that patients with CNV who develop an RPE tear may benefit from initiation of brolucizumab therapy. Additionally, switch to brolucizumab is a valid option in these patients if the disease process seems unresponsive to primary therapy with other anti-VEGF agents, such as aflibercept (Figure 1).

## Data Availability

All data are available upon request to the corresponding author.
